# Supplementary figures and images for: Evolution, persistence, and host adaption of a gonococcal AMR plasmid that emerged in the pre-antibiotic era
Source: PLoS Genet. 2023 May 15;19(5):e1010743. doi: 10.1371/journal.pgen.1010743 (PMC10212123; doi:10.1371/journal.pgen.1010743)

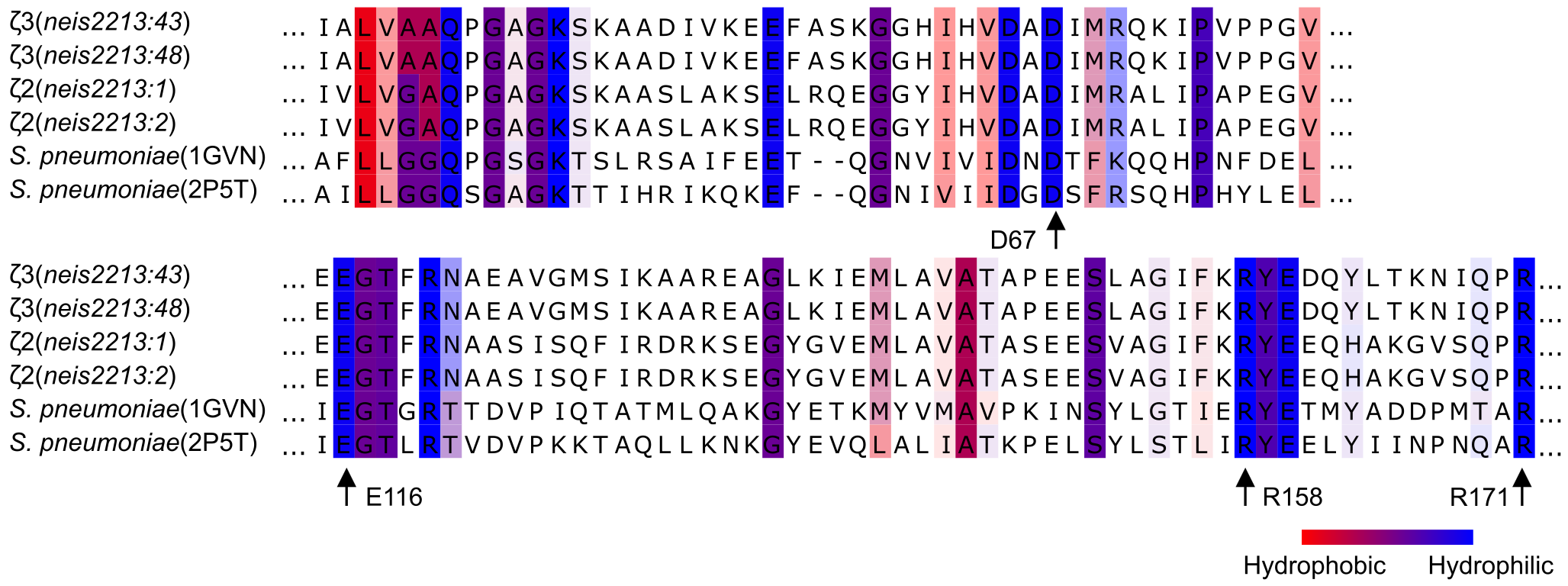

Supplement: S1 Fig — The two most prevalent protein variants of each ζ3 and ζ2 (with their respective neis2213 alleles in brackets) were used for the alignment. Two other characterised Streptococcus pneumoniae ζ proteins were included in the alignment (PDB numbers, 1GVN and 2P5T, are in brackets). Shading intensity is proportionate to percentage conservation. All ζ protein variants have conserved active site residues (catalytic residue and the corresponding amino acid on 1GVN is indicated by the arrow). (TIF) [file pgen.1010743.s001.tif]

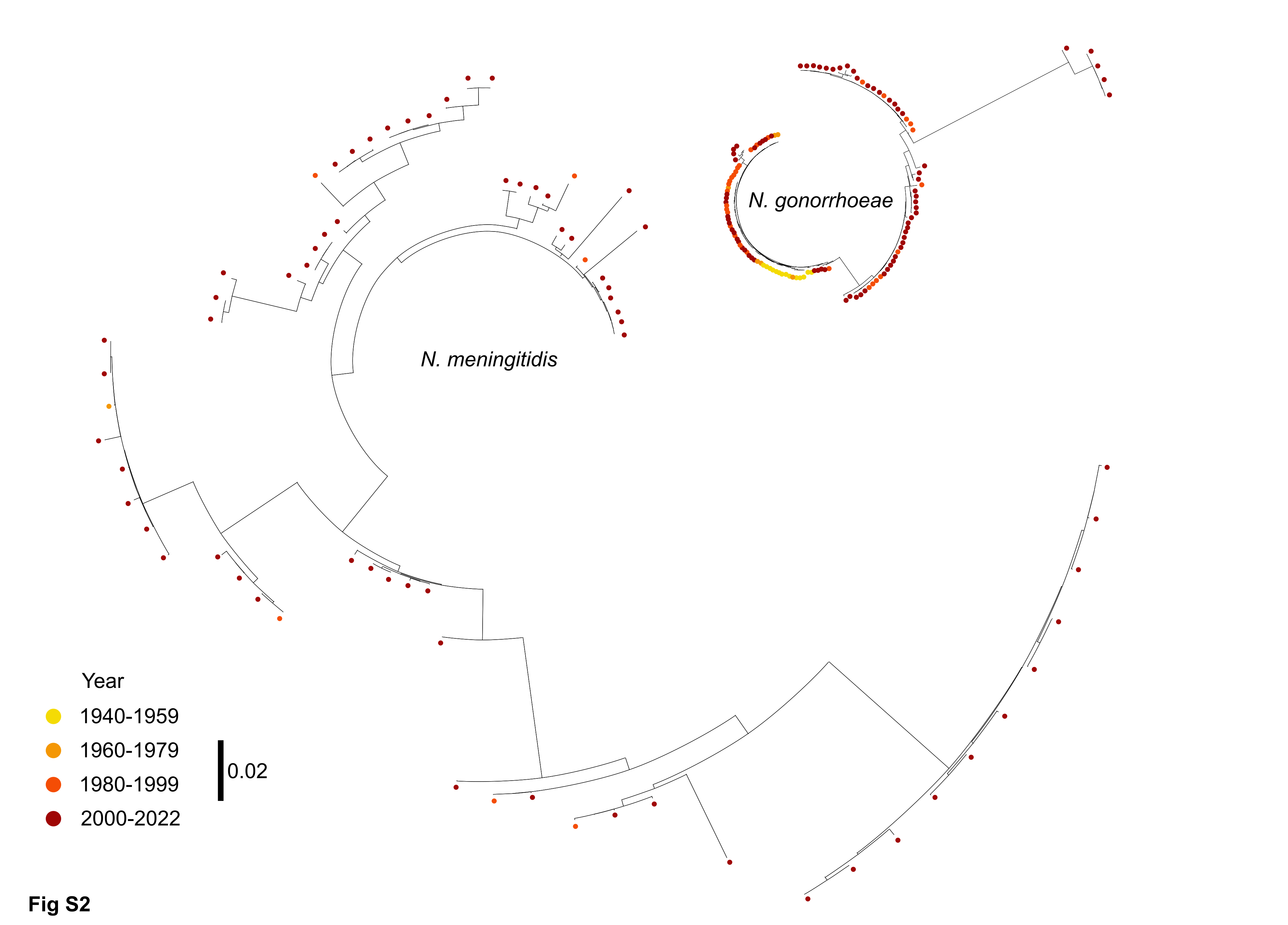

Supplement: S2 Fig — Phylogenetic tree of N. meningitidis and N. gonorrhoeae pConj drawn separately but at the same scale. Each dot represents an isolate, colour-coded according to year of its isolation. (TIF) [file pgen.1010743.s002.tif]

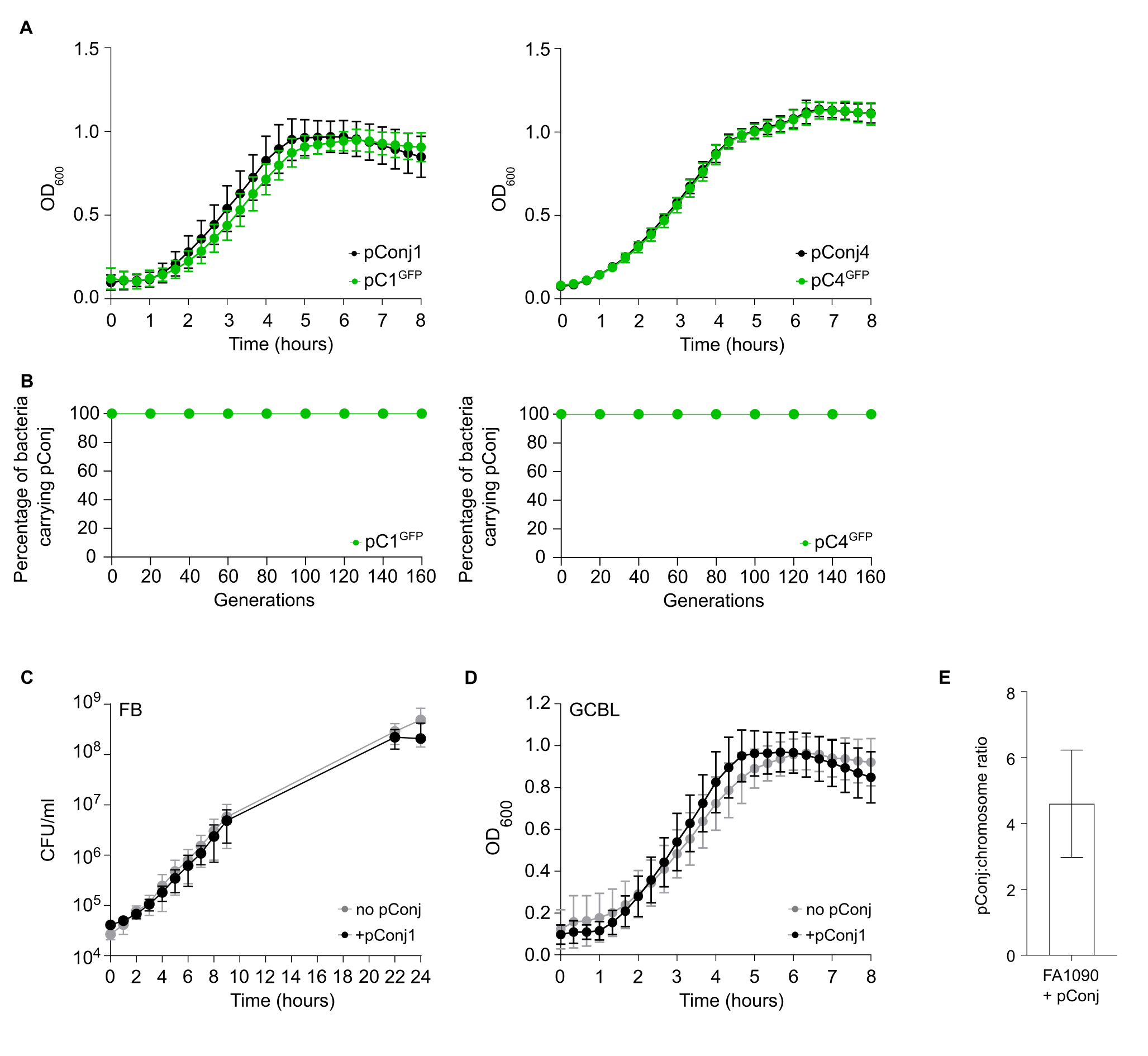

Supplement: S3 Fig — (A) gfp:kan cassette on pConj does not affect growth rates. FA1090 carrying the respective plasmids were first grown overnight before liquid culture was set up using gonococcal base media (GCBL) and monitored over 8 hours. (B) No pConj loss was observed with FA1090 carrying pC1GFP or pC4GFP. FA1090 +/- pConj grows at the same rate in (C) fastidious broth and (D) GCBL over 24 and 8 hours respectively. (E) Ratio of pConj to chromosome is 4.60 ±1.63, as determined by qPCR of res in pConj and recA on the chromosome using bacteria grown to mid-log phase. Results of three independent experiments were analysed with two-way ANOVA with Sidak’s multiple comparisons and shown as mean ± SD. ns, p > 0.05. (TIF) [file pgen.1010743.s003.tif]

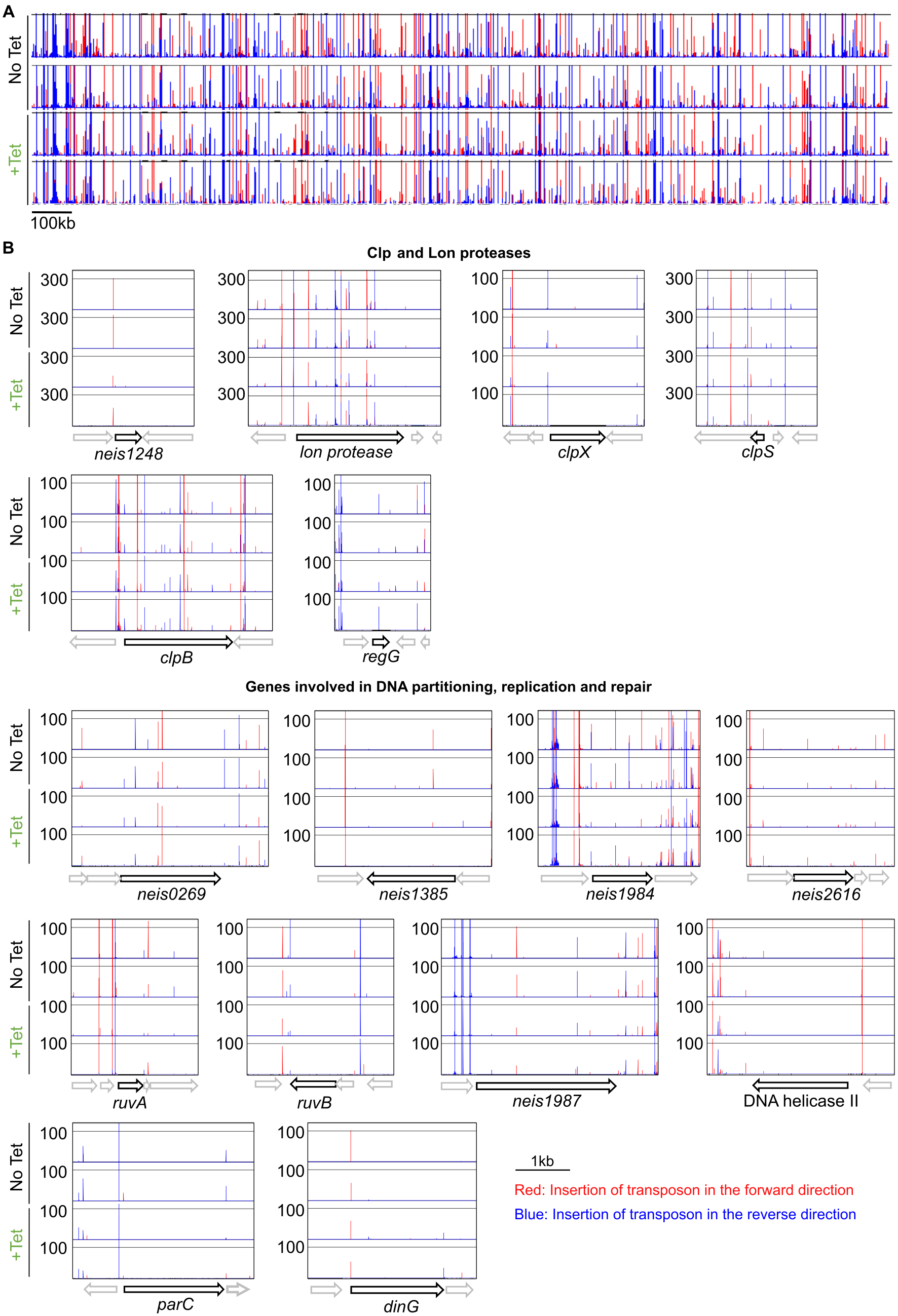

Supplement: S4 Fig — (A) Insertions in the control (kanamycin) and +Tet (kanamycin+tetracycline) conditions were similar in the 56th generation. (B) The profiles of 16 genes with more than one unique insertion, and with functions associated with other genes previously identified to be involved in plasmid maintenance in other bacteria, are shown here, after 16 generations of growth; gene orientation is shown. Gene annotations are consistent with NEIS annotations in PubMLST. No significant differences between control and test conditions were observed. (TIF) [file pgen.1010743.s004.tif]

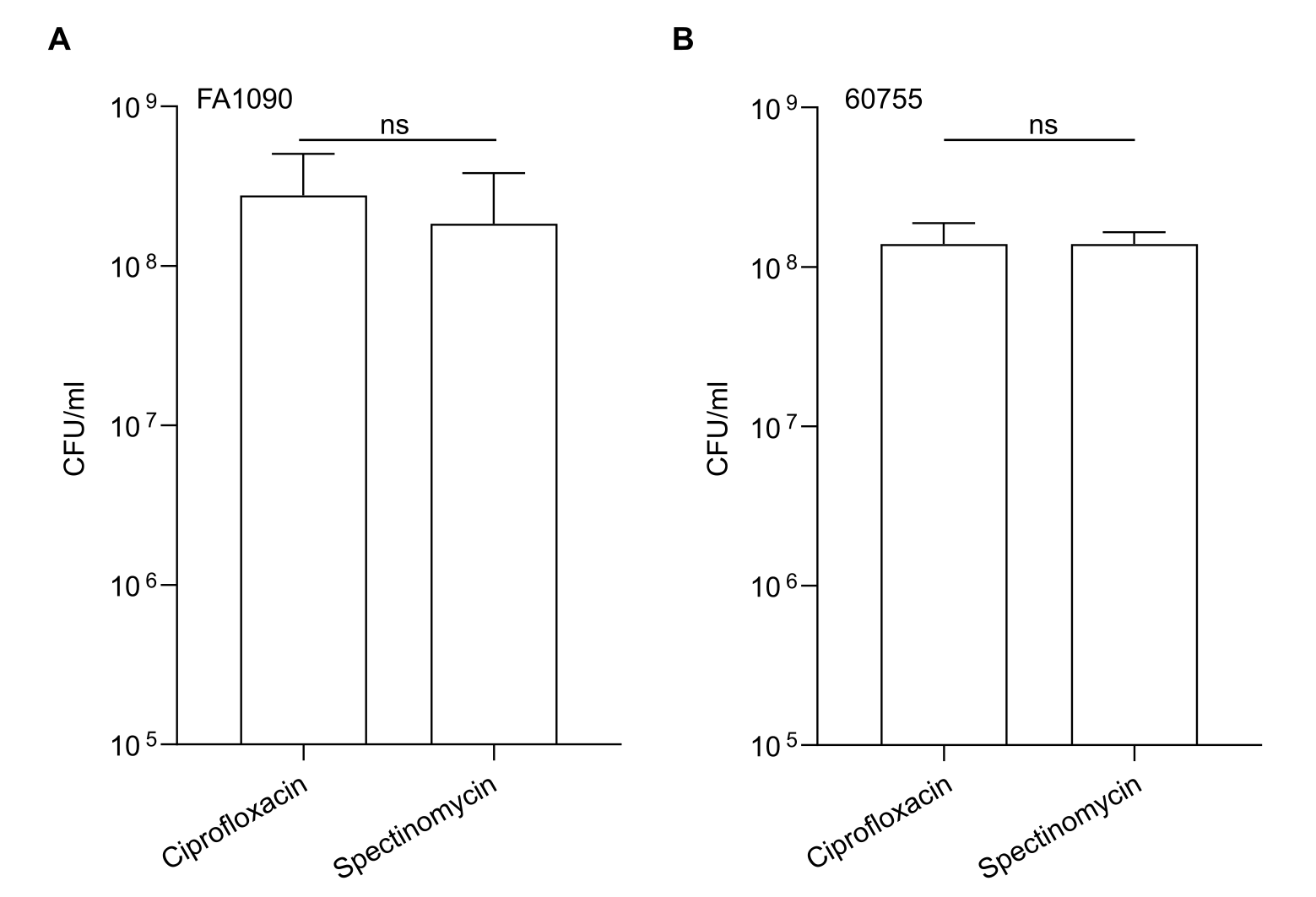

Supplement: S5 Fig — Bacteria were grown overnight on GCB before incubation in 0.5 MIC of ciprofloxacin or spectinomycin for A) FA1090 and B) 60755. Cultures were serially diluted and 5 μl was spotted on GCB agar to determine CFU at the end of the experiment. (TIF) [file pgen.1010743.s005.tif]
