# Supplementary material for: Evolution, persistence, and host adaption of a gonococcal AMR plasmid that emerged in the pre-antibiotic era
Source: PLoS Genet. 2023 May 15;19(5):e1010743. doi: 10.1371/journal.pgen.1010743 (PMC10212123; doi:10.1371/journal.pgen.1010743)
Supplement: S5 Table — Available to view or download from https://figshare.com/s/d2dbf0d466e893028bcf (DOCX) [file pgen.1010743.s011.docx]

**S5 Table. List of strains used in this study.**

| **Strain** | **Background strain** | **Year** | **Country** | **Reference** |
| --- | --- | --- | --- | --- |
| FA1090 | - | 1981 | USA | Gift, Professor Ann Jerse |
| 60755 | - | 2020 | Kenya | [1] |
| 55496 | - | 2020 | Kenya | [1] |
| **Strains for pConj stability assays** | |  |  |  |
| pC1^GFP^ | FA1090 | - | - | This paper |
| pC4^GFP^ | FA1090 | - | - | This paper |
| **Strains for pConj maintenance assays** | |  |  |  |
| 60755 without pConj | 60755 | - | - | This paper |
| pConj1 | FA1090 | - | - | This paper |
| FA1090∆*neis1066::ery*  + pC1^GFP^ | FA1090 | - | - | This paper |
| FA1090∆*neis1845::ery*  + pC1^GFP^ | FA1090 | - | - | This paper |
| FA1090∆*neis2592::ery*  + pC1^GFP^ | FA1090 | - | - | This paper |
| FA1090∆*pilD::ery*  + pC1^GFP^ | FA1090 | - | - | This paper |
| pC∆GL | FA1090 | - | - | This paper |
| pCt | FA1090 | - | - | [2] |
| pCt^GFP^ | FA1090 | - | - | This paper |
| pCtΔ*vapD* | FA1090 | - | - | This paper |
| pCt:*ζ1^K115A^* | FA1090 | - | - | This paper |
| pCtΔ*ζ2* | FA1090 | - | - | This paper |
| pCtΔ*vapD*Δ*ζ1* | FA1090 | - | - | This paper |
| pCtΔTA | FA1090 | - | - | This paper |
| pCtΔTAΔ*parB* | FA1090 | - | - | This paper |
| pCtΔ*parB* | FA1090 | - | - | This paper |
| **Strains for toxicity assay** | |  |  |  |
| pBAD-empty | DH5α | - | - | [3] |
| pBAD-*vapD^pConj^* | DH5α | - | - | This paper |
| pBAD-*vapD^pConj^vapX^pCryp^* | DH5α | - | - | This paper |
| pBAD-*vapD^pConj^vapX^Nm^* | DH5α | - | - | This paper |

References:

1. Cehovin A, Jolley KA, Maiden MCJ, Harrison OB, Tang CM. Association of *Neisseria gonorrhoeae* plasmids with distinct lineages and the economic status of their country of origin. J Infect Dis. 2020;222: 1826–1836.

2. Jones RA, Yee WX, Mader K, Tang CM, Cehovin A. Markerless gene editing in *Neisseria gonorrhoeae*. Microbiology (N Y). 2022;168.

3. Custodio R, Ford RM, Ellison CJ, Liu G, Mickute G, Tang CM, et al. Type VI secretion system killing by commensal *Neisseria* is influenced by expression of type four pili. Elife. 2021;10: 1–31.
