# Supplementary material for: Evolution, persistence, and host adaption of a gonococcal AMR plasmid that emerged in the pre-antibiotic era
Source: PLoS Genet. 2023 May 15;19(5):e1010743. doi: 10.1371/journal.pgen.1010743 (PMC10212123; doi:10.1371/journal.pgen.1010743)
Supplement: Attachment — Submitted filename: Responses to the reviewers final.docx [file pgen.1010743.s013.docx]

**Responses to the reviewers**

**Reviewer #1:**

We are grateful for the reviewer’s positive comments about our manuscript.

*Author summary: I think lines 56-58 might be a bit opaque to the casual reader. Also, I think the authors should make clear when they state that " ... no plasmid loss detected" on line 55, what this means specifically, as it is stated towards the bottom that pConj loss was observed following the introduction of FLQ treatment at the population level.*

We apologise for any misunderstanding. For clarity, we have added that ‘*no plasmid loss detected* after 160 generations of growth in the laboratory’ (line 56), and ‘*mirroring the reduction in pConj carriage* in the gonococcal population…’ (line 62).

We have made other minor changes to the Author Summary to keep the word count below 200.

*Results (and methods): On page 8, the authors describe the distribution on pConj in Neisseria spp. I dont think the methods section describes this part very well. Was pubMLST used for this as well?*

A new section has been added to the Materials and Methods (line 585 et seq.) to clarify the methods we used including the use of pubMLST.

*Results (and methods): I think the authors could spend a few sentences backing up the claim that mobC+repA are 100% diagnostic of pConj presence. Is there any uncertainty around how these genes are detected/annotated on pubMLST?*

We have included the details requested in the new section in Materials and Methods (line 585, see response above). We used two genes to detect both pConj (*traM/neis2241* and *trbC/neis2267*) and pCryp (*mobC/neis2951* and *repA/neis2952*); the selected genes are annotated in pubMLST and are diagnostic as they are present in all variants of these plasmids (Cehovin *et al*.). We have clarified this in the Materials and Methods of our revised manuscript

*Fig2 and associated text in the paper: Perhaps the authors could consider whether any bias in sequencing efforts could affect the estimated fraction of tetM+ pConj over time? I know from painful experience that gonococcal genome sequences are not generated in a completely random fashion and resistant isolates are often overrepresented. TetR might not generate too much fuzz these days, but could tetM+ pConj be associated with resistance to other drugs such as azithromycin or 3rd gen cephalosporins? Could this affect estimates? I dont think there are any easy answers to this, but it might be worthwhile giving this some thought.*

We agree with the reviewer that sequence databases are inherently biased and can never be fully representative. As cephalosporin resistance is polygenic so more difficult to interpret, we examined whether there is a correlation between pConj and azithromycin resistance. The presence of the following 23S rRNA alleles were considered when determining azithromycin resistance: 2599T (2611T in *E. coli*) and 2143G (2059G in *E. coli*) Chisholm *et al*., and Ng *et al*.).

Results demonstrate that isolates that are resistant to azithromycin are less likely to carry pConj (odds ratio 0.09907, 95% confidence interval: 0.05470 – 0.1767). This suggests that our estimates of the prevalence of pConj are not biased by associated resistance to azithromycin.

In line with this and reviewer 3’s comment, we now discuss the limitation of current databases, and the need to expand collections (line 355-357).

*Finally, I think it would be good if the authors could share metadata on presence/absence of plasmids and gene elements across Neisseria species, in the form of a csv file or similar.*

As requested, these data now included these data to Table S1 which now contains all relevant metadata.

**Reviewer #2:**

We are grateful for the reviewer’s positive comments about our manuscript. The reviewer raises six specific points

1. *Whether the plasmid is conserved as stated is hard to discern from the data presented. Can the changes be represented graphically (Figure 1) to present the reader with a way to judge the conservation level? Are the other plasmids of similar size or chromosomal loci that can be compared to determine whether there is, or is not, a high level of conservation?*

We thank the reviewer for raising this point, as the only way to judge the degree of conservation in Figure 1 was scale bar we provided. In line with their suggestion, we analysed the conservation of all available pConj sequences from *Neisseria meningitidis* (n = 70 with good quality sequence). The marked diversity of pConj in the meningococcus (additional Figure S2) compared with its conservation in the gonococcus highlights the point clearly. We have amended our manuscript to describe this analysis (lines 154-157).

2. *The authors provide no analysis to model how the tetracycline resistance determinant was acquired or other plasmid changes.*

Previous publications have proposed how *tetM* was acquired, so we did not include them in our article. In line with the reviewer’s comment, we discuss the acquisition of *tetM* in our revised manuscript (line 346-348, two additional references).

3. *The analysis of plasmid stability in the TnSeq approach is confusing and possibly incomplete. Why was the representation of transposon insertions in the population decreasing over generations? This reduction in the insertion representation could be due to the growth differences of different mutants.*

We agree. This is supported by the consistency of data from four independent passages (Figure S3).

*Still, it is difficult to believe this would not occur in the first few generations as opposed to over so many generations. These data raise the question of whether the population was sampled properly.*

The reduction in library size is evident in early generations as well as later. The number of mutants in our initial library was 183,919, at the 8^th^ generation was 85,496 (average of four biological replicates), and by the 16^th^ generation was 62,841. This would predict that the number of mutants would be around 13,500 mutants by the 56^th^ generation while we found there was around 5-6,000 left.

The consistency of data from independent passages shows that the library was adequately sampled at each passage (at least 1.5 x 10^9^ CFU were used to start each passage) and when bacteria were collected for sequencing (when we sampled at least 3 x 10^9^ CFU). At both these steps the library size (~ 184,000 mutants) was exceeded by at least four orders of magnitude.

*Finally, since this is a conjugative plasmid, the role of conjugal transfer in repopulating the cells that lost the plasmid was not considered.*

The reviewer raises a good point which we considered. In preliminary experiments, we did not see appreciable conjugation in liquid media. At a 1:1 donor:recipient ratio, the conjugation rate was ~ 0.26% (*i.e.* approximately 2 in 1000 recipients acquired the plasmid) during 24 hrs growth so plasmid transfer could occur in our experiments, but at a very low rate. We now mention this in our discussion (line 363-365).

4. *The conclusion that pConj remains in the population after the use of tetracycline ceased is an important conclusion. It would be worth clarifying the timelines of isolation and parameters of tetracycline, especially since two countries with different health recommendations are involved as is some countries where antibiotics may not be regulated. Perhaps adding a timeline under Figure XX would clarify the antibiotic history and put the US, UK, and other isolates in perspective. Is it clear that there was no longer tetracycline use after the recommendations changed?*

It is not clear to which figure the reviewer is referring; we have specific timelines in Figure 2 which contains country specific data. In contrast, Figure 6D is a generic representation of what has happened to the plasmid (with no mention of country-specific policies and thence dates); we prefer to not add dates to Figure 6.

Although tetracycline was no longer used to treat gonococcal infection after the dates indicated in the text (line 164 and 172) and Figure 2, individuals with gonorrhoea may be given tetracyclines for treating chlamydia (pointed out by reviewer 3). We acknowledge this is as a potential confounding factor in our manuscript (line 354 -358), but highlight that the persistence of the markerless plasmid provides evidence for the tenacity of pConj.

5. *Figure 3 presents negative data and could be moved to the supplement. The conclusion that some identified chromosomal genes are false positives is premature. This conclusion should be supported by constructing the loss-of-function mutants and directly testing their role in plasmid maintenance.*

The lack of insertions in candidates means they are likely to be false positives. However, in line with the reviewer’s comments, we have generated the following mutants:

∆*neis1066*: highest logCPM with a negative logFC and significant *q*-value

∆*neis1845* and ∆*neis2592*: two hits with the most significant *q*-value

∆*pilD*: control

No mutant showed any plasmid loss indicating the TIS only gave false positive hits. The construction of the mutants and results are described in lines 209-214 and 517-526.

6. *The Meyer lab adapted the pConj-tet plasmid for the Hermes gene delivery system that can introduce genes into transformation noncompetent cells. This reference could be cited, perhaps in the introduction (Kupsch et al. Mol Gen Genet 250:558 1996).*

We are aware of this work. We are not sure where to mention this in our paper.

**Reviewer #3:**

We are grateful for the reviewer’s positive comments about our manuscript.

Abstract
Line 33: *“pConj confers tetracycline resistance”. It does when it carries the tetM gene, but some strains don’t. Would be better to say “pConj can confer tetracycline resistance”.*

Thank you, We have added the word ‘can’ as suggested (line 32).

Results
Line 143: *I think it is important to specify throughout the text that the dates of emergence of each plasmid are actually dependent on the collections we have, i.e. the first isolate from the collection with a pConj was from 1940, but it doesn’t mean that it was not present before. It is not present in isolates from 1928-1939 from the collection the authors are using but these old isolates are very scarce and all from a Danish study (Golparian et al, 2020).*

We thank the reviewer for this comment, and agree with them about the limitations of current collections. We have added this to the Discussion of our revised manuscript (line 356-358).

Lines 143-149: *I assume the authors state that Group I pConj appeared first because it includes the oldest isolates. However, how is the pConj tree in Fig1D rooted? From that tree, Group I pConj appears to emerge from Group II. It looks unrooted, I suggest rooting this tree so it is visually/phylogenetically more evident that Group I emerged first.*
The tree was not rooted as we are unaware of an unrelated comparator that could be used to root the tree. We tried to track the evolutionary history of pConj with BEAST but this was unable to identify an origin/timescale, mainly due to the conservation of pConj in the gonococcus. However, our tree has been constructed with RAXML-NG, updated from RAXML, and constructed the tree 1,000 times which gives a more robust result (see modified lines 570-573).

S1 *Fig legend: please, specify what 1GVN stands for.*

This refers to the PBD number in structural databases. We now include this in the corresponding legend.

Lines 170-171: *my understanding is that tetracyclines are not the first choice for uncomplicated gonorrhea, but doxycycline is used in certain (sometimes quite often) circumstances (doi: 10.1177/0956462420949126) as well as to treat other STIs. I do not think doxycycline has been completely absent from treatment since 2004. A background sporadic usage of tetracyclines may have allowed the tetM pConj plasmid to stay in the gonococcus population even after the removal of tetracyclines as first line treatment.*

Please see our response to reviewer 2, question 4.

Line 308: *the authors could also check if these results are consistent with the genomic data that they first use from PubMLST. They could easily detect mutations leading to ciprofloxacin resistance (in gyrA, parC – at least gyrA S91F) and assess whether these isolates carry pConj. This would be a way of re-confirming their results in a big clinical dataset.*

We thank the reviewers for their suggestion. However, ciprofloxacin resistance is not the same as exposure to ciprofloxacin: for example, progeny of a ciprofloxacin-resistant strain will also carry the gene encoding for ciprofloxacin resistance even they have never been exposed to ciprofloxacin.

Furthermore, pConj could be acquired, lost or re-acquired after a strain has become ciprofloxacin resistant.

Therefore, we feel that the analysis of pConj carriage in ciprofloxacin resistant versus ciprofloxacin sensitive strains could be misleading.

**References**

Cehovin A, Jolley KA, Maiden MCJ, Harrison OB, Tang CM (Feb 2020). Association of Neisseria gonorrhoeae plasmids with distinct lineages and the economic status of their country of origin. J Infect Dis pii: jiaa003. doi: 10.1093/infdis/jiaa003.

Chisholm SA, Dave J, Ison CA (Sep 2010). High-level azithromycin resistance occurs in *Neisseria gonorrhoeae* as a result of a single point mutation in the 23S rRNA genes. Antimicrob Agents Chemother. doi: 10.1128/AAC.00309-10.

Ng LK, Martin I, Liu G, Bryden L (Sep 2002). Mutation in 23S rRNA associated with macrolide resistance in *Neisseria gonorrhoeae*. Antimicrob Agents Chemother. doi: 10.1128/AAC.46.9.3020-3025.2002.
